# Normal fasting triglyceride levels and incident type 2 diabetes in the general population

**DOI:** 10.1186/s12933-022-01530-8

**Published:** 2022-06-18

**Authors:** Tamas Szili-Torok, Stephan J. L. Bakker, Uwe J. F. Tietge

**Affiliations:** 1 Department of Internal Medicine, University Medical Center Groningen, Groningen, The Netherlands; 2 Division of Clinical Chemistry, Department of Laboratory Medicine (LABMED), Karolinska Institutet, H5, Alfred Nobels Alle 8, S‐141 83 Stockholm, Sweden; 3 Clinical Chemistry, Karolinska University Laboratory, Karolinska University Hospital, SE-141 86 Stockholm, Sweden

**Keywords:** Triglycerides, Diabetes, De novo, Dyslipidemia, Cohort study

## Abstract

**Background:**

Type 2 diabetes is increasing worldwide. Traditionally, only hypertriglyceridemia is considered a risk factor. We investigated whether also normal triglycerides prospectively associate with incident type 2 diabetes in healthy subjects.

**Methods:**

Incident type 2 diabetes was determined in healthy individuals with normal triglyceride levels from a prospective longitudinal cohort study (PREVEND, n = 2085, 11.4-year median follow-up).

**Results:**

Type 2 diabetes incidence was 3.8%. In linear regression analysis baseline insulin, HOMA-IR, total cholesterol, HDL cholesterol, eGFR, systolic blood pressure (all p < 0.001), glucose, age and creatinine (all p < 0.01) independently associated with triglycerides within the normal range, comparable to what would be expected from associations with increased triglycerides. In Kaplan-Meier analysis sex-stratified tertiles of normal triglycerides prospectively associated with de novo type 2 diabetes (p < 0.001). Cox regression confirmed a significant prospective association independent of HOMA-IR [HR (95% CI), 1.39 (1.12, 1.74), p = 0.002] and several other recognized risk factors.

**Conclusions:**

Even in healthy subjects without metabolic syndrome increasing triglyceride levels within the normal range confer a continuous increase in type 2 diabetes incidence. These data indicate that virtually everyone could potentially benefit from triglyceride lowering, further encouraging implementation of lifestyle changes in the general population.

**Supplementary information:**

The online version contains supplementary material available at 10.1186/s12933-022-01530-8.

## Background

Type 2 diabetes is a common disease with worldwide rising incidence and prevalence [1]. Affected patients experience a substantial increase in morbidity and mortality translating not only into individual suffering but also significant health care costs [2]. Better and earlier identification of individuals at risk is required, particularly in the light of the potential success of lifestyle interventions to delay type 2 diabetes onset [3]. In the cardiovascular field, triglyceride levels currently receive increasing attention as biomarker of dyslipidemia, with hypertriglyceridemia (above 150 mg/dL) being gradually associated with adverse outcomes [4]. Regarding incident type 2 diabetes, triglycerides have mainly been considered in the context of hypertriglyceridemia as component of the metabolic syndrome [5, 6]. In such studies participants with normal triglycerides are commonly used as reference group. Whether circulating triglycerides within the normal range and in the absence of the metabolic syndrome also confer a risk of de novo type 2 diabetes is currently not known. Therefore, the present study investigated in healthy participants of a large general population cohort whether variation of plasma triglycerides within the normal range is associated with incident type 2 diabetes.

## Methods

The Prevention of Renal and Vascular End-stage Disease (PREVEND) study is a prospective longitudinal cohort study from the Northern Netherlands [7]. PREVEND was approved by the medical ethics committee of the University Medical Center Groningen (MEC96/01/022) and carried out according to the Declaration of Helsinki. All participants provided written informed consent. Individuals between 28 and 75 years of age were invited to participate. Pregnant women and subjects using insulin were excluded leaving 8592 participants for the initial screening (1997–1998). Five thousand seven hundred and sixty-nine participants had complete data with respect to type 2 diabetes follow-up and triglyceride concentrations and of these a total of 5665 subjects were diabetes free at baseline. Next, we excluded subjects with existing diabetes (definition from the American Diabetes Association) or metabolic syndrome (definition of NHLBI/American Heart Association) at baseline [8], then participants with fasting triglycerides > 150 mg/dL (n = 2163 remaining). Please note that HbA1c levels were not available, since this was not standard of care in the Netherlands at the time when PREVEND was established. While anti-hypertensive medication was allowed, participants using lipid modulating medication (such as statins, fibrates, fish oil or niacin) were excluded (n = 78) leaving a final number of n = 2085 for the current analysis. A comparison table between included and excluded individuals is shown in the Additional file 1: Table S1. A schematic outline of scheduled follow-up visits in PREVEND is given in Additional file 1: Fig. S1.

### Outcomes and end point

Regarding outcome measures, the main predictor was plasma triglycerides, the end point incident type 2 diabetes defined as per protocol (i) fasting plasma glucose ≥ 7.0 mmol/L, (ii) non-fasting glucose ≥ 11.1 mmol/L, (iii) self-report of a type 2 diabetes diagnosis or (iv) initiation of glucose lowering medication (data retrieved from a central pharmacy registry). Please note that no case of self-reported type 2 diabetes diagnosis occurred in the present study.

### General patient characteristics

Participants of PREVEND attended 2 outpatient visits during which baseline data was collected including general parameters such as demographics, lifestyle measures including alcohol use and smoking, anthropometric measurements, family history of type 2 diabetes, medical history and medication use. Longitudinal information regarding medication was obtained from the hospital-pharmacy dispensing registry which had complete information on more than 95% of the participants. Hypertension was recorded based on either information provided by patients, physician diagnosis, the use of antihypertensive medication or a blood pressure measurement of ≥ 140/90 mmHg. Body mass index (BMI) was calculated by dividing weight (kg) by height squared (m^2^).

### Laboratory measurements

Fasting venous plasma was obtained after an overnight fast. Triglycerides were measured enzymatically (Abbott Laboratories, Abbott Park, IL, United States), fasting plasma glucose and total cholesterol by dry chemistry (Eastman Kodak, Rochester, NY, USA), insulin with immunoturbidimetry (Diazyme Laboratories, Poway, CA, USA) and urinary albumin using nephelometry (Dade Behring Diagnostic, Marburg, Germany). HDL cholesterol was assessed utilizing a homogenous method (direct HDL; Aeroset System; Abbott Laboratories, Abbott), serum creatinine by an enzymatic method (Roche Diagnostics, Mannheim, Germany) and serum cystatin C by immunoassay (Gentian AS, Moss, Norway). High sensitivity C-reactive protein was measured by nephelometry (Dade Behring Diagnostic). The methods did not change during inclusion and all measurements took place in the accredited routine clinical chemistry laboratory of the University Medical Center Groningen (UMCG).

The estimated glomerular filtration rate was calculated using the Chronic Kidney Disease Epidemiology Collaboration (CKD-EPI) combined creatinine cystatin C equation [135 × min(Scr/κ, 1)α × max(Scr/κ, 1) − 0.601 × min(Scys/0.8, 1) − 0.375 × max(Scys/0.8, 1) − 0.711 × 0.995Age [× 0.969 if female] [× 1.08 if black], where Scr is serum creatinine and Scys is serum cystatin C] [9]. Homeostatic model assessment of insulin resistance was calculated with the following formula: [fasting plasma insulin (mU/L) × fasting plasma glucose (mmol/L)]/22.5. LDL cholesterol concentrations were determined using the Friedewald equation (all participants had triglycerides levels below 150 mg/dL) as, LDL cholesterol = total cholesterol – HDL cholesterol – (triglycerides / 5).

### Statistical analysis

Statistical analysis was carried out using R Studio (RStudio Team, 2020). P-values below 0.05 were considered significant. χ^2^-test was used for categorical variables [total numbers (%)], one way ANOVA for normally distributed variables (mean ± SD) and Kruskal-Wallis test for skewed continuous variables (median [interquartile range, IQR]). Multivariate linear regression with backward elimination was performed to test the association of baseline variables with triglycerides. Kaplan–Meier curves were constructed based on tertiles of triglycerides, significance explored using log-rank testing. Prospective associations between baseline triglycerides and incident type 2 diabetes were explored with Cox regression analyses using models based on known type 2 diabetes risk factors and baseline characteristics significantly associated with triglycerides as specified. Schoenfeld residuals test showed that the proportional hazard assumption was not violated. In order to visualize the prospective association between continuous normal triglyceride concentrations and adjusted hazard ratios of type 2 diabetes, a graph was constructed using log_2_ transformed triglycerides adjusted for age and sex. Log_2_ transformation was chosen instead of spline terms as AIC scores showed a better model fit. In addition, subgroup analyses with interaction tests were used to determine hazard ratios (HR) across categories of baseline characteristics using median values as cutoff for continuous variables.

## Results

Out of the 2085 included participants, 70 (3.4%) developed type 2 diabetes during a median follow-up of 11.4 [10.9–12.2] years. Incident type 2 diabetes during follow-up increased gradually from low to high tertiles of normal triglycerides (n = 13 for low, n = 17 for medium and n = 40 for high, Table 1, p < 0.001). With increasing tertiles of triglycerides, subjects were significantly older, had higher BMI and waist circumference (median [IQR] for men was 91.0 [85.0–97.5] cm and 79.0 [73.0–85.9] cm for women) as well as glucose, insulin, HOMA-IR and hsCRP values (Table 1). Further, total and LDL cholesterol levels were higher, while HDL cholesterol was lower. Blood pressure increased with increasing tertiles of triglycerides, and kidney function was significantly lower, although of note participants were healthy and recorded values largely within the normal range. In decreasing order of strength, linear regression analysis identified a similar set of baseline parameters independently associated with triglycerides within the normal range as expected from associations with raised triglycerides [10–12], namely insulin, HOMA-IR, total cholesterol, HDL cholesterol, eGFR, age, creatinine, systolic blood pressure, and waist circumference (Table 2).

**Table 1:** Baseline characteristics according to tertiles of fasting triglyceride levels

Variable	Low tertile (n = 705)	Medium tertile (n = 685)	High tertile (n = 695)	P value
Triglycerides (mg/dL)	57.6 [49.5–65.5]	82.4 [77.1–89.5]	116 [105.8–130.2]	< 0.001
Diabetes mellitus type II diagnosis (%)	1.8	2.9	5.5	< 0.001
Age (years)	43.3 [37.1–52.9]	47.2 [39.1–56.4]	50.7 [42.8–60.8]	< 0.001
Sex (% female)	57.7	57.2	57.4	0.982
Intoxication				
Alcohol use (%)	75.5	76.8	78.4	0.422
Past smoker (%)	53.5	55.3	56.0	0.622
Constituents of the metabolic syndrome				
BMI (kg/m^2^)	23.8 [21.9–26.1]	25.0 [23.0–27.3]	26.0 [24.2–28.0]	< 0.001
Waist circumference (cm)	80.5 [73.5–89.0]	84.0 [77.0–92.0]	88.0 [81.0–96.5]	< 0.001
Plasma glucose (mmol/L)	4.6 [4.3–4.9]	4.6 [4.3–5]	4.7 [4.4–5.1]	< 0.001
Plasma insulin (mlU/L)	5.9 [4.5–8.0]	7.1 [5.2–9.6]	8.5 [6.4–11.6]	< 0.001
HOMA-IR	1.2 [0.9–1.7]	1.4 [1–2]	1.8 [1.3–2.4]	< 0.001
hsCRP (mg/L)	0.6 [0.3–1.4]	0.9 [0.4–2.1]	1.3 [0.6–2.6]	< 0.001
Total cholesterol (mg/dL)	189.5 [170.1–212.7]	208.8 [184.1–233.2]	220.4 [197.4–247.5]	< 0.001
HDL cholesterol (mg/dL)	59.9 [50.7–70.4]	55.7 [45.6–66.5]	50.7 [42.9–60.7]	< 0.001
LDL cholesterol (mg/dL)	123.3 [106–146.5]	142.9 [121.7–166.8]	160.3 [135.2–186.2]	< 0.001
Systolic blood pressure (mmHg)	117.0 [109.0–129.0]	121.0 [111.0–134.0]	127.0 [116.0–140.0]	< 0.001
Diastolic blood pressure (mmHg)	70.0 [63.0–76.0]	71.0 [66.0–78.0]	74.0 [68.0–80.0]	< 0.001
Renal function				
Serum creatinine (mmol/L)	69.6 [61–77.2]	69.6 [61–79.3]	70.7 [62.1–80.4]	0.022
eGFR (mL/min/1.73 m^2^)	103.0 [91.7–111.4]	99.3 [89.5–108.6]	94.8 [84.1–106]	< 0.001
Urinary albumin concentration (mg/L)	5.5 [3.7–9.5]	5.9 [3.7–10.8]	6.1 [3.5–11.4]	0.168
Medication use				
Antihypertensives use (%)	6.1	8.9	11.4	= 0.002

Normally distributed continuous variables are presented as mean ± SD, skewed continuous variables are expressed as median [IQR], categorical data are given as n (%). In order to evaluate the presence of statistically significant differences between the tertiles, χ^2^-test was used for categorical variables, Kruskall-Wallis test for skewed variables and one way ANOVA for variables with a normal distribution

**Table 2:** Multivariate linear regression with backward elimination

Final model after backward elimination		
Variable	Standardized beta [95% CI]	P value
Insulin	0.52 [0.29, 0.74]	< 0.001
HOMA-IR	− 0.37 [− 0.60, − 0.12]	< 0.001
Total cholesterol	0.37 [0.33, 0.41]	< 0.001
HDL cholesterol	− 0.32 [− 0.36, − 0.28]	< 0.001
eGFR	− 0.20 [− 0.27, − 0.14]	< 0.001
Glucose	0.05 [− 0.01, 0.11]	0.102
Systolic blood pressure	0.09 [0.05–0.14]	< 0.001
Waist circumference	0.09 [0.0, 0.10]	0.023
Age	− 0.11 [− 0.17, − 0.05]	< 0.001
Creatinine	− 0.10 [− 0.16, − 0.05]	< 0.001

**Fig. 1:**
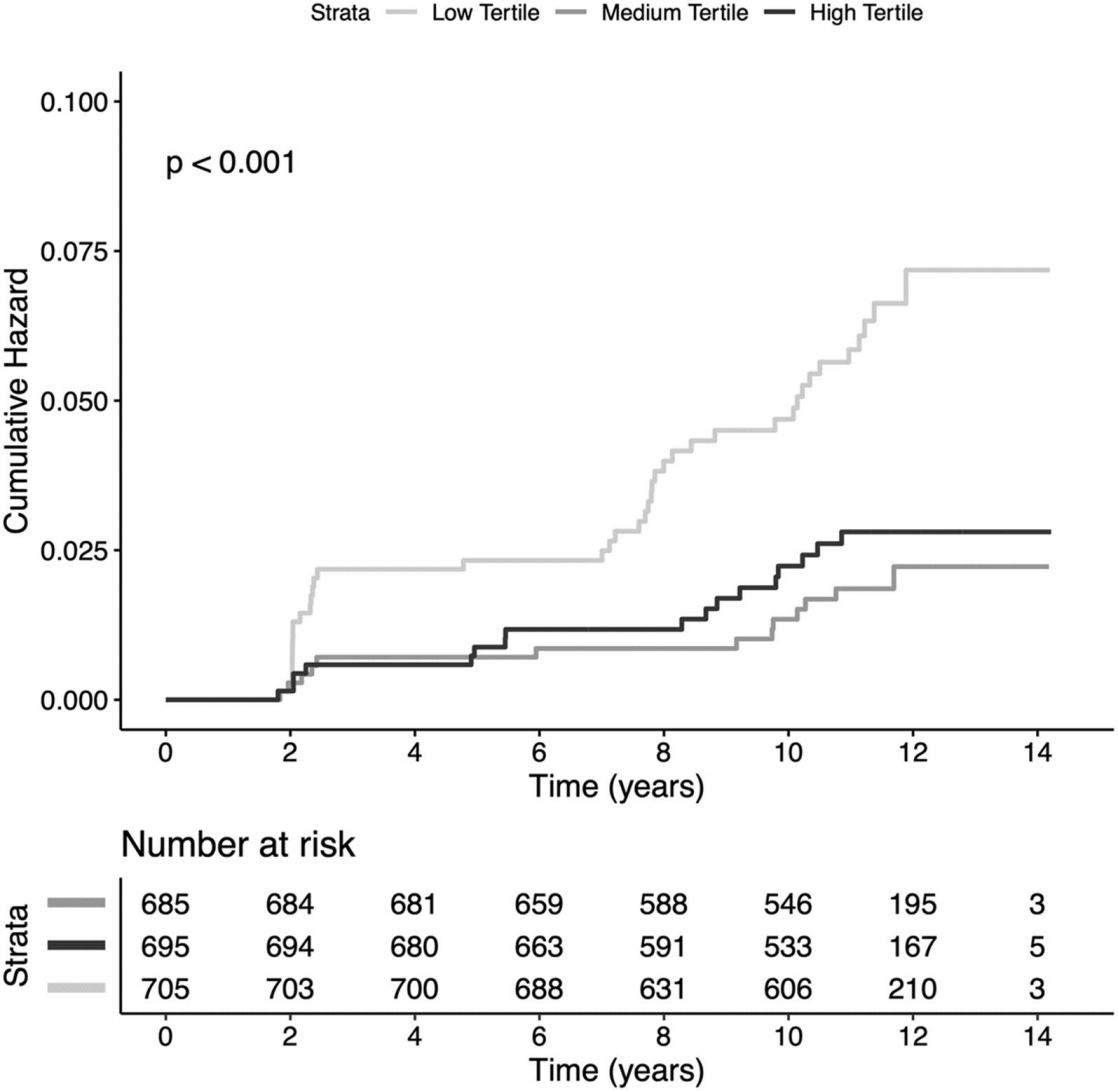
Kaplan-Meier analysis depicting type 2 diabetes according to tertiles of triglycerides (log rank test, p value < 0.001)

**Fig. 2:**
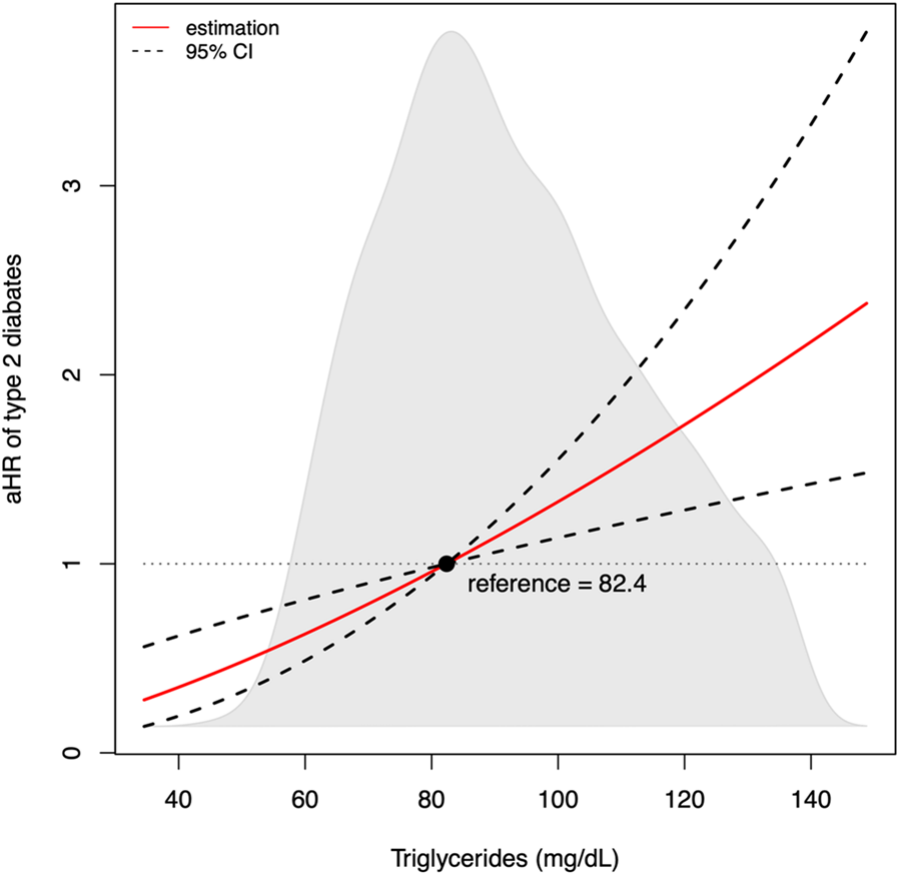
Relative risk of type 2 diabetes development according to triglyceride levels. Relative risk was obtained by Cox regression analysis using log_2_ transformed triglycerides, adjusted for age and sex. The reference value is the median of plasma triglycerides in this cohort (82.4 mg·dL)

**Fig. 3:**
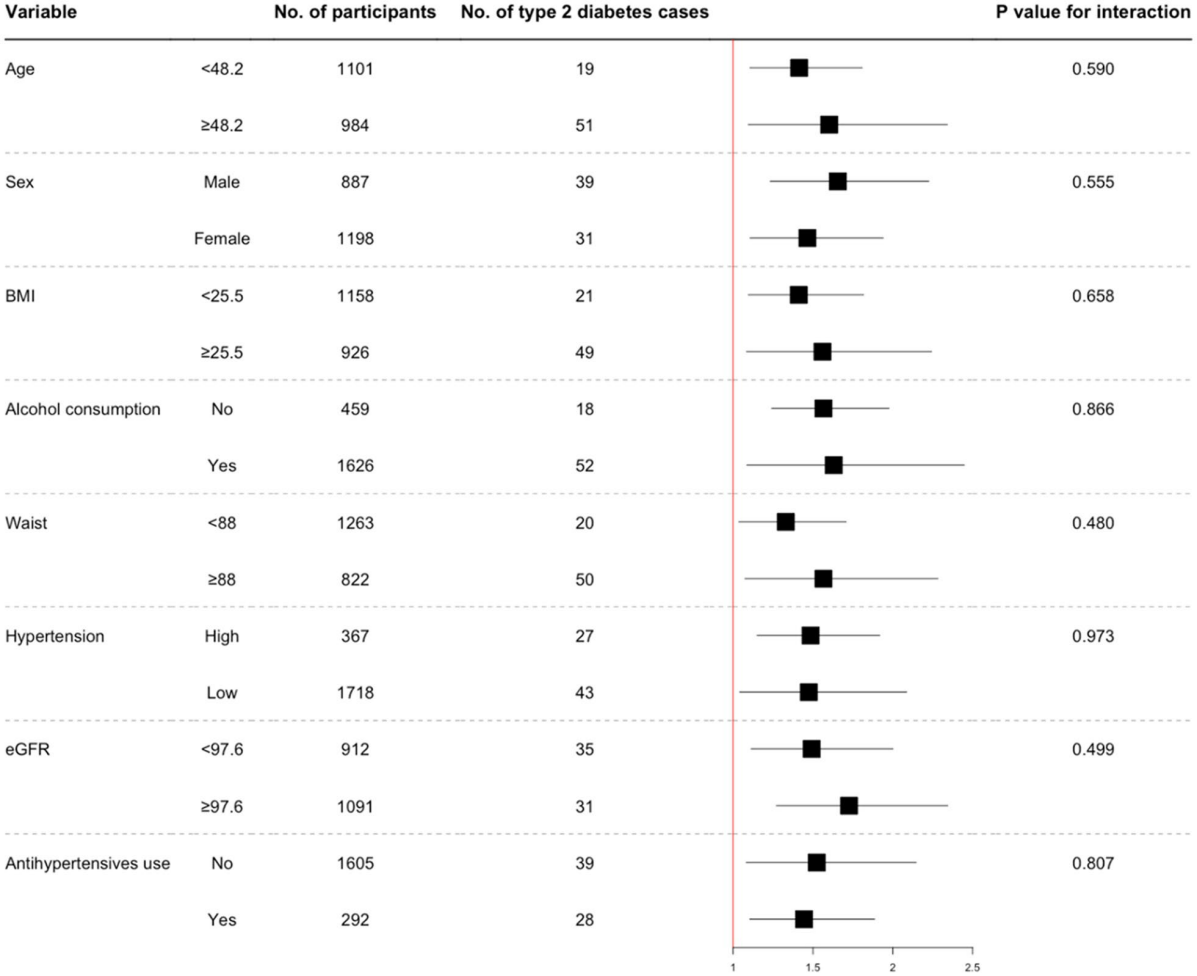
Hazard ratios for incident type 2 diabetes per 10 mg/dL increase in normal plasma triglyceride values by several participant-level characteristics. HR (95% CI) for the association of baseline triglyceride concentrations with incident type 2 diabetes were obtained using Cox regression analysis. Age is given in years, BMI, body mass index (kg/m^2^); waist is given in cm; eGFR, estimated glomerular filtration rate (mL/min/1.73 m^2^). Hypertension was defined as a systolic blood pressure > 140 mmHg

In Kaplan–Meier analysis increasing tertiles of triglycerides were significantly associated with higher incident type 2 diabetes (Fig. 1, p < 0.001). Furthermore, triglyceride concentrations were significantly prospectively associated with type 2 diabetes development in Cox regression analyses (Table 3, model 1, HR 1.58 [1.29, 1.94], p < 0.001). Addition of age and sex (model 2, aHR, 1.48 [1.20, 1.83], p < 0.001) to model 1 did not weaken this association. After adjustment for BMI, waist circumference, systolic and diastolic blood pressure (model 3, aHR, 1.26 [1.01, 1.58], p = 0.040) and HOMA-IR, total cholesterol and hs-CRP (model 4, aHR, 1.29 [1.02, 1.63], p = 0.031) higher normal triglyceride concentrations remained significantly associated with incident type 2 diabetes. Finally, adjusting for renal function (model 5, aHR, 1.54 [1.24, 1.91], p < 0.001) and medication use (model 6, aHR, 1.48 [1.20, 1.83], p < 0.001) did not materially change the conclusions reached from the previous models. The aHR of type 2 diabetes continuously increased with increasing normal triglyceride concentrations (Fig. 2). There were no significant differences detected in the HR of normal triglyceride levels across several clinically relevant participant level characteristics notably including sex (Fig. 3). The hazard ratio for participants in the PREVEND cohort (n = 613) who had hypertriglyceridemia was 1.04 [1.00–1.09] (p = 0.075).

**Table 3:** Association of normal triglycerides with incident type 2 diabetes in the general population

	(a) HR [95% CI]	P value
Model 1		
Crude analysis	1.58 [1.29, 1.94]	< 0.001
Model 2		
Adjusted for age and sex	1.48 [1.20, 1.83]	< 0.001
Model 3		
Model 2 + BMI, waist circumference, systolic blood pressure and diastolic blood pressure	1.26 [1.01, 1.58]	0.040
Model 4		
Model 2 + HOMA-IR total cholesterol, hs-CRP	1.29 [1.02, 1.63]	0.031
Model 5		
Model 2 + creatinine, eGFR and urinary albumin	1.54 [1.24, 1.91]	< 0.001
Model 6		
Model 2 + antihypertensive medication use	1.48 [1.20, 1.83]	< 0.001

## Discussion

The results of this study demonstrate that even in healthy subjects from the general population an increase in plasma triglycerides within the currently handled normal range translates into a continuous increase in incident type 2 diabetes. Interestingly, all participating subjects had normal glucose values and were free of metabolic syndrome. Still, similar associations between triglycerides and other parameters such as BMI, HOMA-IR, HDL cholesterol or eGFR were observed as typically seen in hypertriglyceridemic patients with the metabolic syndrome [10–12]. These data indicate that the association between plasma triglycerides and those biomarkers is very dynamic and basically extends throughout the full range of values. In a clinical perspective these observations could indicate that, similar to the recent lowering of the upper normal values for blood glucose levels, also a re-evaluation of the normal range of plasma triglycerides might be worth considering. However, before taking such an approach independent confirmation in other cohorts ideally including different ethnicities and socio-economical settings would be desirable. Furthermore, it might be valuable to explore potential mechanisms how triglycerides affect insulin resistance and beta cell function by conducting experimental studies in humans as well as preclinical models, where suitable.

PREVEND is a large general population study with sufficient statistical power and a very thorough follow-up. However, it consists of predominantly White participants from the North of the Netherlands. Another limitation of our study is that HbA1c levels are not available as biomarker to establish a diagnosis of type 2 diabetes and that data on sleep, physical activity or nutrition were not recorded. By selecting for participants with normal triglycerides only, a potential impact of this selection bias that was introduced on purpose cannot be excluded. Further it is worth pointing out that only a single measurement of plasma triglycerides at the time of inclusion into the study was taken.

## Conclusions

In conclusion, this work demonstrates that in the absence of the metabolic syndrome in healthy subjects increasing triglyceride levels within the normal range confer a continuous increase in type 2 diabetes incidence. With respect to prevention or intervention our results indicate that potentially every subject could benefit from lifestyle improvements [3] such as increased aerobic exercise, weight loss as well as decreased consumption of sucrose- or fructose-sweetened beverages and alcohol [13] with the goal to keep circulating triglyceride levels as low as possible.

## Supplementary Information

**Additional** **file** **1**: **Table S1.** Baseline characteristics table of included and excluded individuals in the current study from the PREVEND cohort. **Figure S1.** Summary of visits of the PREVEND study (years are median values among all participants).

## Data Availability

The dataset analyzed during the current study is available from the corresponding author upon reasonable request.
