# Supplementary material for: Normal fasting triglyceride levels and incident type 2 diabetes in the general population
Source: Cardiovasc Diabetol. 2022 Jun 18;21:111. doi: 10.1186/s12933-022-01530-8 (PMC9206357; doi:10.1186/s12933-022-01530-8)

**Online supplement to:**

**Normal fasting triglyceride levels and incident type 2 diabetes in the general population**

Tamas Szili-Torok, MD^1^, Stephan J. L. Bakker, MD, PhD^1^, Uwe J. F. Tietge, MD, PhD^2,3^

^1^Department of Internal Medicine, University Medical Center Groningen, Groningen, The Netherlands

^2^Division of Clinical Chemistry, Department of Laboratory Medicine, Karolinska Institutet, Stockholm, Sweden

^3^Clinical Chemistry, Karolinska University Laboratory, Karolinska University Hospital, SE-141 86 Stockholm, Sweden

**Supplemental Table S1:** Baseline characteristics table of included and excluded individuals in the current study from the PREVEND cohort.

|  | | **Excluded** (n=3686) | **Included** (n=2085) | **P value** |
| --- | --- | --- | --- | --- |
| Triglycerides (mg/dL) | | 121.3 [83.3 - 180.7] | 82.4 [64.7 - 105.4] | <0.001 |
| Age (years) | | 48.6 [40.0 - 58.5] | 47.4 [39.0 - 57.4] | 0.002 |
| Sex (% Female) | | 44.7 | 57.5 | <0.001 |
| Alcohol use (%) | | 27.9 | 23.1 | <0.001 |
| Smoking in the past (%) | | 77.5 | 54.9 | <0.001 |
| BMI (kg/m2) | | 25.8 [23.3 - 28.8] | 25.1 [22.9 - 27.2] | <0.001 |
| Waist circumference (cm) | Female | 83.0 [74.1 - 93.0] | 79.0 [ 73.0 - 85.9 ] | <0.001 |
|  | Male | 94.0 [86.0 - 101.5] | 91.0 [85.0 - 97.5] | <0.001 |
| Glucose (mmol/L) | | 4.7 [4.4 - 5.2] | 4.6 [4.3 - 5.0] | <0.001 |
| Insulin (mlU/L) | | 8.4 [5.7 - 13.0] | 7.1 [5.2 - 9.6] | <0.001 |
| HOMA-IR | | 1.8 [1.1 - 3.0] | 1.5 [1.0 - 2.0] | <0.001 |
| hsCRP (mg/dL) | | 1.4 [0.6 - 3.2] | 0.9 [0.4 - 2.1] | <0.001 |
| Total cholesterol (mmol/L) | | 219.3 [189.5 - 250.3] | 205.0 [181.7 - 232.0] | <0.001 |
| HDL cholesterol (mmol/L) | | 45.6 [37.9 - 56.1] | 55.3 [46.4 - 65.7] | <0.001 |
| LDL cholesterol (mmol/L) | | 158.9 [129.4 - 188.8] | 141.8 [117.1 - 167.8] | <0.001 |
| Systolic blood pressure (mmHg) | | 126.0 [115.0 - 140.0] | 122.0 [111.0 - 135.0] | <0.001 |
| Diastolic blood pressure (mmHg) | | 74.0 [68.0 - 80.0] | 71.0 [66.0 - 78.0] | <0.001 |
| Serum creatinine concentration  (mmol/L) | | 71.8 [62.1 - 81.5] | 69.6 [61.0 - 79.3] | 0.003 |
| eGFR (mL/min/1.73m^2) | | 96.5 [84.7 - 107.2] | 99.3 [88.0 - 108.9] | <0.001 |
| Urinary Albumin output | | 7.1 [4.1 - 13.5] | 5.8 [3.6 - 10.6] | <0.001 |
| Lipid lowering medication (% ) | | 8.1 | 0 | <0.001 |
| Antihypertensive medication use (% ) | | 19.5 | 8.8 | <0.001 |

Skewed continuous variables are expressed as median [IQR], categorical data are given as n (%). In order to evaluate the presence of statistically significant differences between the tertiles, χ^2^-test was used for categorical variables and Wilcoxon signed rank test for skewed variables.

**Supplemental figure S1:** Summary of visits of the PREVEND study (years are median values among all participants).


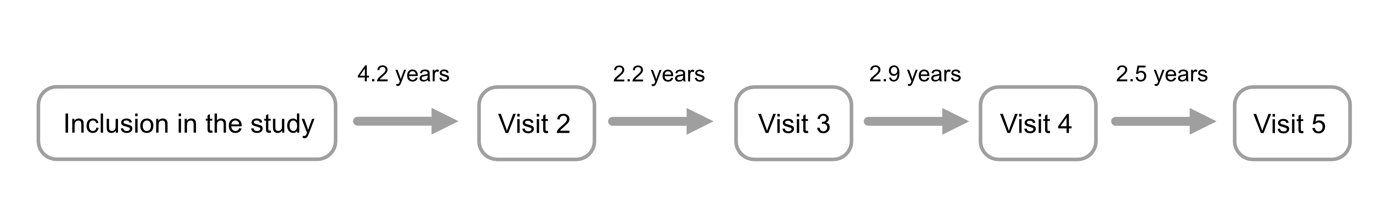

Supplement: Supplementary file 1 — Additional file 1: Table S1. Baseline characteristics table of included and excluded individuals in the current study from the PREVEND cohort. Figure S1. Summary of visits of the PREVEND study (years are median values among all participants). [file 12933_2022_1530_MOESM1_ESM.docx]
